# Supplementary material for: The genome sequence of the Dark Spectacle, Abrostola triplasia (Linnaeus, 1758)
Source: Wellcome Open Res. 2023 Jun 27;8:278. [Version 1] doi: 10.12688/wellcomeopenres.19624.1 (PMC10521094; doi:10.12688/wellcomeopenres.19624.1)
Supplement: Peer review history

# Reviewer report: Reviewer response for version 1

**DOI:** 10.21956/wellcomeopenres.21740.r63622

**Published:** 2023-09-12

**Authors:** William Hemstrom

**Affiliations:** 1 University of California, California, USA

Boyes *et al.* present here a chromosome-level genome assembly for *Abrostola triplasia*, the first such for the species. The reference individual was sequenced using a mix of PacBio long reads and assembled with the aid of Hi-C data. They then annotated the genome using transcriptomic data. The result is a very high quality genome, available from the ENA.

 I have no major issues with this work or the author's methodology.

 Minor Issues:

1. The y axis label for Figure 3 is uninformative, and should probably be replaced with something like "Coverage".
2. The grey line on Figure 4 is not visible behind the teal line for the most part. It should probably be re-plotted with the gray line as the top-most graphical layer.
3. Figure 5 needs axis labels and a color legend. While I understand that the axes denote chromosomes and positions along them, labels would be helpful.
4. There is at least one position shown on Figure 5 that seems to have a globally high interaction frequencies to the rest of the genome. This should probably be addressed briefly in the text.
5. The assembly section thoroughly describes the tools used, but should probably more fully describe the parameter choices and options used in each step. If this would make the text too long for publication here, these details could be relegated to a supplementary document. Alternatively (and probably preferably), the scripts used to conduct the analysis could be made available.
6. The second sentence of the Background section reads a bit awkwardly. Perhaps something like "... there are relatively few records from Scotland, mostly from southern parts of the region."?

As a side-note, I very much appreciate the careful documentation of tool versions in Table 3.

Are sufficient details of methods and materials provided to allow replication by others?

Yes

Is the rationale for creating the dataset(s) clearly described?

Yes

Are the datasets clearly presented in a useable and accessible format?

Yes

Are the protocols appropriate and is the work technically sound?

Yes

Reviewer Expertise:

I am a population and ecological geneticist specializing on methodological approaches. My major study species include Monarch Butterflies. I am not a specialist on genome assemblies, and so am not the best person to dive deeply into the technicalities of that aspect. However, the overall quality of the genome produced seems excellent in comparison to what I typically work with.

I confirm that I have read this submission and believe that I have an appropriate level of expertise to confirm that it is of an acceptable scientific standard.

# Reviewer report: Reviewer response for version 1

**DOI:** 10.21956/wellcomeopenres.21740.r65939

**Published:** 2023-09-12

**Authors:** Jesper Bechsgaard, Jeppe Bayer Pedersen

**Affiliations:** 1 Aarhus University, Aarhus, Denmark

This data note describes the reference genome of *Abrostola triplasia. *The analyses are sound and well described. I am sure that this data can be used for exciting analyses of decline and inter-specific competition with the *A. tripartita*. If the authors could outline some of the questions this data could be used to address in the Background section, instead of saying 'A genome sequence for *Abrostola triplasia* will contribute to a growing data set of resources for understanding lepidopteran biology', which does not really say anything, it would be very interesting.

Are sufficient details of methods and materials provided to allow replication by others?

Yes

Is the rationale for creating the dataset(s) clearly described?

Partly

Are the datasets clearly presented in a useable and accessible format?

Yes

Are the protocols appropriate and is the work technically sound?

Yes

Reviewer Expertise:

Population genetics, molecular evolution

We confirm that we have read this submission and believe that we have an appropriate level of expertise to confirm that it is of an acceptable scientific standard.
